# Simulation, In Vitro, and In Vivo Cytotoxicity Assessments of Methotrexate-Loaded pH-Responsive Nanocarriers

**DOI:** 10.3390/polym13183153

**Published:** 2021-09-17

**Authors:** Mahmood Barani, Mohammad Reza Hajinezhad, Saman Sargazi, Mahira Zeeshan, Abbas Rahdar, Sadanand Pandey, Mehrdad Khatami, Farshid Zargari

**Affiliations:** 1 Medical Mycology and Bacteriology Research Center, Kerman University of Medical Sciences, Kerman 7616913555, Iran; Mahmoodbarani7@gmail.com; 2 Basic Veterinary Science Department, Veterinary Faculty, University of Zabol, Zabol 9861335856, Iran; hajinezhad@uoz.ac.ir; 3 Cellular and Molecular Research Center, Research Institute of Cellular and Molecular Sciences in Infectious Diseases, Zahedan 9816743463, Iran; 4 Department of Pharmacy, Faculty of Biological Sciences, Quaid-i-Azam University, Islamabad 45320, Pakistan; mz1712@yahoo.com; 5 Department of Physics, University of Zabol, Zabol 9861335856, Iran; 6 Department of Chemistry, College of Natural Science, Yeungnam University, 280 Daehak-Ro, Gyeongsan 38541, Korea; 7 Noncommunicable Diseases Research Center, Bam University of Medical Sciences, Bam 76617-71967, Iran; mehrdad7khatami@gmail.com; 8 Department of Medical Biotechnology, Faculty of Medical Sciences, Tarbiat Modares University, Tehran 14115-111, Iran; 9 Pharmacology Research Center, Zahedan University of Medical Sciences, Zahedan 9816743463, Iran; farshid6694@gmail.com; 10 Department of Chemistry, Faculty of Science, University of Sistan and Baluchestan, Zahedan 98135674, Iran

**Keywords:** methotrexate, niosome, pH-responsive, in-vitro, in-vivo, simulation

## Abstract

In this study, pH-responsive niosomal methotrexate (MTX) modified with ergosterol was prepared for potential anticancer application. The prepared formulation had a size of 176.7 ± 3.4 nm, zeta potential of −31.5 ± 2.6 mV, EE% of 76.9 ± 2.5%, and a pH-responsive behavior in two different pHs (5.4 and 7.4). In-silico evaluations showed that MTX intended to make a strong hydrogen bond with Span 60 compartments involving N2 and O4 atoms in glutamic acid and N7 atom in pteridine ring moieties, respectively. The cytotoxic effects of free and pH-MTX/Nio were assessed against MCF7 and HUVECs. Compared with free MTX, we found significantly lower IC50s when MCF7 cells were treated with niosomal MTX (84.03 vs. 9.464 µg/mL after 48 h, respectively). Moreover, lower cell killing activity was observed for this formulation in normal cells. The pH-MTX/Nio exhibited a set of morphological changes in MCF7 cells observed during cell death. In-vivo results demonstrated that intraperitoneal administration of free MTX (2 mg/kg) after six weeks caused a significant increase in serum blood urea nitrogen (BUN), serum creatinine, and serum malondialdehyde (MDA) levels of rats compared to the normal control rats. Treatment with 2 and 4 mg/kg doses of pH-MTX/Nio significantly increased serum BUN, serum creatinine, and serum lipid peroxidation. Still, the safety profile of such formulations in healthy cells/tissues should be further investigated.

## 1. Introduction

Methotrexate (MTX) is classified as an anti-folate and anti-metabolite drug. It is a well-known chemotherapeutic and immunosuppressive agent [1]. Common therapeutic uses encompass treatment of autoimmune diseases, blood cancer, and tumors of breast cancer, osteosarcoma, lymphoma, etc. [1,2,3]. Mechanistically, MTX inhibits the synthesis of purines and pyrimidines involved in producing DNA and RNA of rapidly dividing cells, thus impeding cancer cell growth and progression [1,2]. Methotrexate and antitumor necrosis factor treatment improve endothelial function in patients with inflammatory arthritis [4]. Another study reported that combination treatment with MTX and α-tocopherol suppressed TNBC cell proliferation. Caspase-3 activation and poly(adenosine diphosphate-ribose) polymerase cleavage were observed in the α-tocopherol succinate/MTX-treated cells [5]. Another study performed on 64 patients showed that continuous low-dose CTX and MTX are minimally toxic and effective in heavily pretreated breast cancer patients [6].

However, MTX has pharmacokinetic issues of lower aqueous solubility (0.01 mg/mL), permeability (log *p* = 0.3), and absorption with ultimate low bioavailability at higher doses [7]. Moreover, its solubility and degradation depend on pH, with minimum degradation in aqueous fluids between pH values of 6.6 and 8.2 at a higher temperature [8]. Furthermore, higher doses are usually required to treat cancer, which is responsible for deleterious side effects, including hepatotoxicity, gastrointestinal problems, lung diseases, fatigue, cytopenia, renal insufficiencies, etc. [1].

Recently, nanotechnology revolutionized medicine and drug delivery and advanced drug therapeutics to a new level [9,10,11,12,13,14,15,16,17]. Nanodrug delivery systems with a size range of 1–100 nm in at least one dimension served as a drug carrier with multiple advantages such as better and prolonged targeting ability, reduced dose, decreased off-site targeting, lower adverse effects and toxicities, enhanced drug solubility, and pharmacokinetic parameters, overcoming drug resistance, sustained drug release, etc. [18,19,20]. Nano-drug delivery systems have multiple types of carriers according to the nature of the drug to achieve therapeutic goals—such as polymeric nanoparticles, lipidic nanocarriers (liposomes, transfersome, etc.), and amphiphilic surfactant carriers (niosomes) [21,22,23,24,25].

To enhance bioavailability and tumor targeting efficiency with lower side effects, low water-solubility drugs such as MTX can be easily entrapped within niosomes. Niosomes are versatile self-assembled non-ionic surfactant-based vesicles stabilized in the presence of cholesterol [22]. Niosomes are completely biocompatible, stable, having both hydrophobic and hydrophilic features to entrap all kinds of drugs [22,26]. Thus, niosomes are suitable nanocarriers to eradicate drug delivery issues, and various drug-loaded niosomes were prepared to treat the cancers in the recent past [27,28,29]. For instance, studies reported encapsulation of MTX inside the niosomal vesicles with good entrapment efficiency and therapeutic output [30,31]. Mostly, conventional niosomes were stabilized by a membrane stabilizer such as cholesterol [32]. However, in our previous study, ergosterol proved to be a better stabilizer than cholesterol and produced smaller niosomes with better polydispersity index and sustained release characteristics [33]. Ergosterol is a sterol found in the fungal cell membrane, functioning just like cholesterol in animal cells. Ergosterol has more double bonds in its structure, which is one of the reasons for its greater interactions with phospholipids that impart structural integrity and stability [34].

Furthermore, it was found that ergosterol naturally converted into vitamin D3 inside mammals [35], and it has been proved that vitamin D3 reduced tumor incidence and progression [36]. Thus, the addition of ergosterol not only improved niosome properties but it may also have positive therapeutic outcomes in cancer. Additionally, niosomes exhibited pH-responsive drug release properties, with a higher amount of drug discharge at an acidic pH and lower at normal physiological pH [27]. The pH-mediated characteristics directed niosomes to target and release the drug inside the tumor cells, with lower pH values compared to cells within a normal physiological pH range.

Therefore, we aimed to develop pH-responsive niosomes with ergosterol stabilizer as a suitable nanocarrier for MTX delivery with improved bioavailability and targeting towards the tumor cells and with reduced off-site targeting to normal cells. As per our knowledge, the niosomes-ergosterol combination has not been used previously for the drug delivery of methotrexate in cancer. Briefly, we have prepared pH-responsive niosomal MTX with Tween 60, Span 60, and ergosterol as a major component along with other ingredients. Then the prepared niosomes were analyzed for physicochemical properties and entrapment efficiency. Furthermore, as a novel approach, we have developed an in silico model of the lipid bilayer composed of niosomal components and ergosterol. The incorporation of molecular dynamics simulations assisted us in studying the interactions of MTX with the lipid bilayer. Afterward, in-vitro drug release studies at different pH and cytotoxic effects were studied at MCF7 and HUVECs cell lines. We have evaluated the cytotoxicity of niosomal MTX on tumor and normal cells in comparison to free MTX. Next, 2 and 4 mg/kg doses of niosomal MTX were intraperitoneally administered to the mice to investigate toxicological effects on the kidney and testis and examine its effects on serological biochemical parameters.

## 2. Materials and Methods

### 2.1. Chemicals and Cell Line

Span 60, Tween 60, ergosterol, cisplatin, 3-(4,5-Dimethylthiazol-2-yl)-2,5-diphenyltetrazolium bromide (MTT), and dimethyl sulfoxide (DMSO) were purchased from Sigma-Aldrich (Steinheim am Albuch, Germany and St Louis, MO, USA). Methotrexate injection (MTX) was supplied from Teva (Irvine, CA, USA). Cholesteryl hemisuccinate (CHEMS) was obtained from Avanti polar, USA. Dulbecco’s modified Eagle medium (DMEM) and fetal bovine serum (FBS) were procured from Gibco (Grand Island, NY, USA). 0.25% trypsin-ethylenediaminetetraacetic acid (EDTA) solution, 1% penicillin-streptomycin solution, trypan blue, and phosphate-buffered saline (PBS) was obtained from INOCLON (Tehran, Iran). The human umbilical vein endothelial cells (HUVECs) and human breast cancer cells (MCF7) were purchased from the cell repository of the Pasteur Institute of Iran (Tehran, Iran). Cells were cultivated in DMEM medium containing 10% heat-inactivated FBS and antibiotic mixture and maintained under standard conditions (95% air, 5% CO2, and 37 °C).

### 2.2. Preparation of pH-Responsive Niosomal MTX

**Scheme 1:**
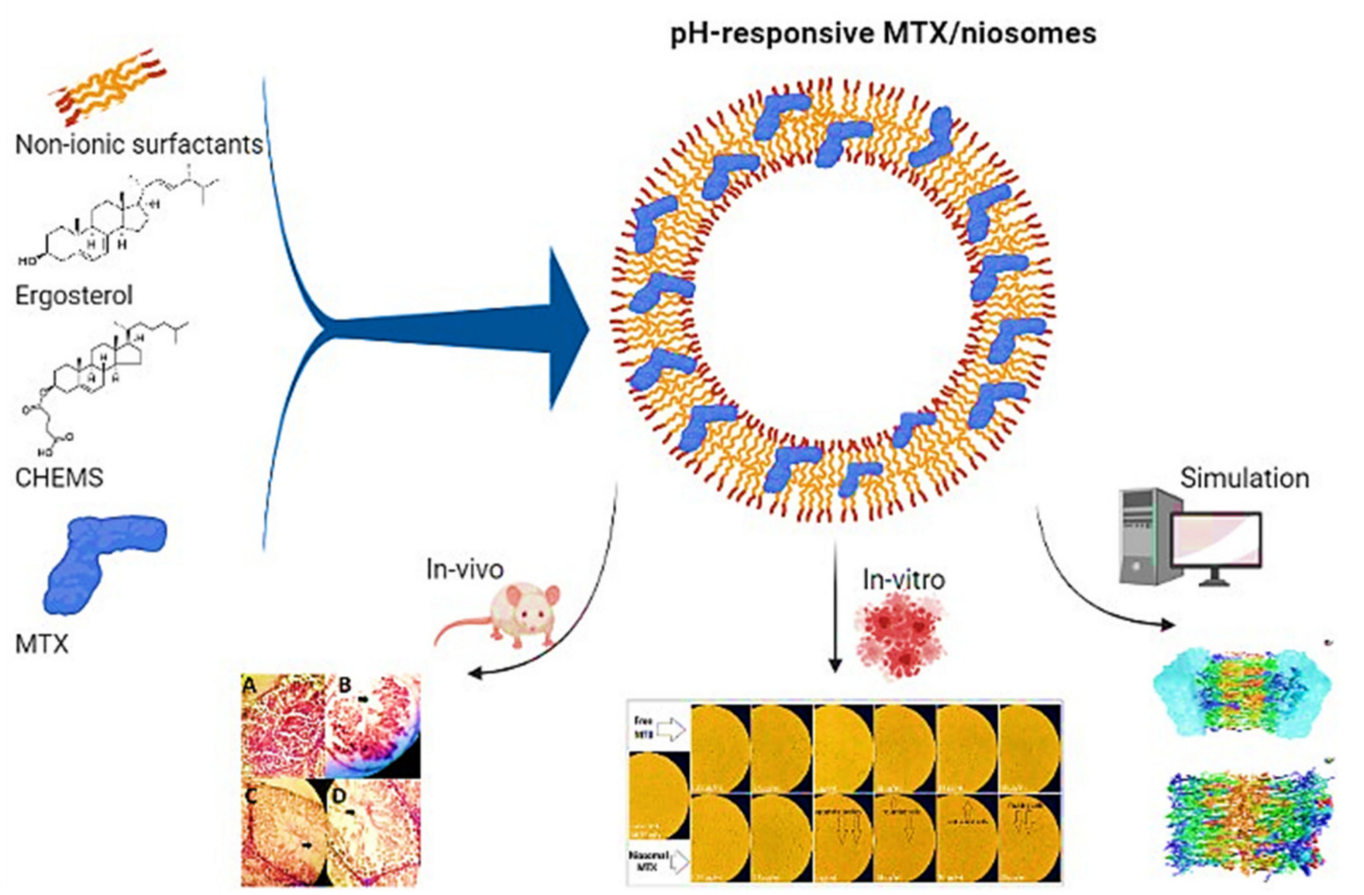
Schematic representation for synthesizing and evaluations of pH-responsive niosomal MTX created with BioRender.com.

Niosomes were made using a slight modification of the thin film hydration method (TFH) employed in previous papers [33,37]. Span 60, Tween 60, ergosterol, and CHEMS were dissolved in 5 mL chloroform and poured into a rotary-flask in a molar ratio of 0.3:0.3:0.3:0.1 (total lipid content of 300 μM). Using a rotary evaporator (Laboroa 4003, Heidolph, Germany), the chloroform was progressively evaporated at 180 rpm and 60 °C under vacuum until a thin, dry layer of the components formed on the inner wall of the flask. The inner lipid film was hydrated with ultrapure water containing 510 ppm of MTX by rotating the flask in a rotary evaporator at 60 °C and 180 rpm to guarantee complete hydration. Centrifugation at 15,000 rpm for 60 min (5415D, Eppendorf, Germany) can separate the unencapsulated MTX from the encapsulated ones. The resultant dispersion was sonicated for 5 min in a bath sonicator (Thermo Fisher Scientific, Waltham, MA, USA) for particle size reduction and homogenization and then double-filtered (0.45 and 0.22 μm pore size, polycarbonate membrane, Sartorius AG, Göttingen, Germany). The niosomal solution was left to mature overnight at 4 °C before being employed for further experiments. A schematic representation for synthesizing and evaluations of pH-responsive niosomal MTX was depicted in Scheme 1.

### 2.3. Simulation

#### System Setup and Simulation Details

**Figure 1:**
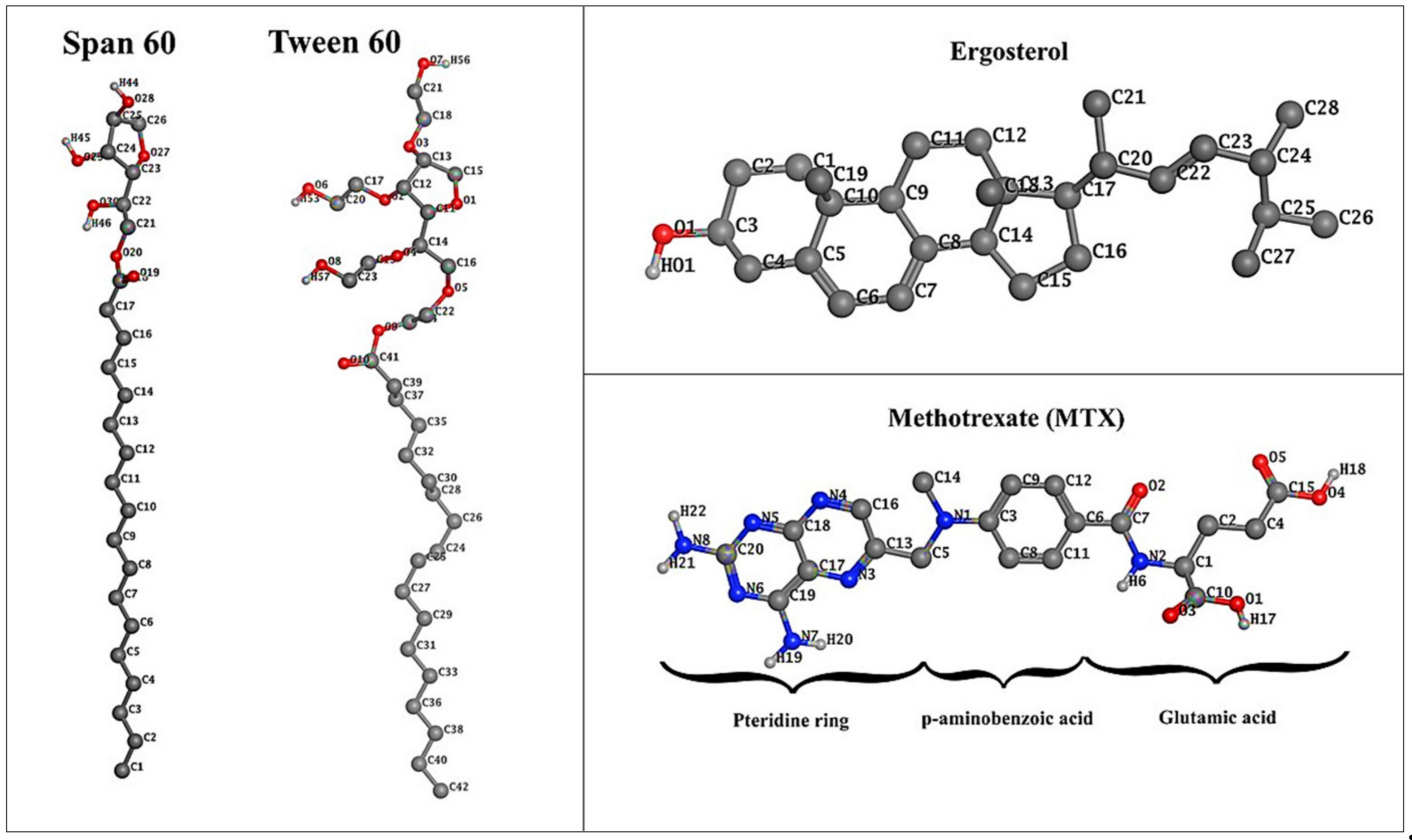
Molecular structures of methotrexate, span 60, Tween 60, and ergosterol with atom numbering are based on the amber nomenclature.

Simulation studies have gained lots of interest in recent years [38,39,40,41,42]. The progress in quantitative models and simulations of biomolecular systems across different scales will increase our understanding of complex biological phenomena [43,44,45]. This allows the integration and interpretation of heterogeneous experiments reporting on the structure and dynamics of biomolecules and enable the prediction of new properties that may not be evident from experiments [46,47]. The structures of Span 60, Tween 60, ergosterol, and methotrexate (MTX) were retrieved from PubChem [48] and then geometrically optimized using B3LYP/6-3G(d,p) calculations. The optimized structures of Span 60, Tween 60, and ergosterol, shown in Figure 1, were applied to build the niosome bilayer model containing a 35:35:30 molar ratio of Span 60:Tween 60:ergosterol by using the CELL microcosmos 2.2 software [49]. The bilayer contains 82 span 60, 80 Tween 60, and 70 ergosterol molecules in each leaflet, leading to 230 molecules in total. Span 60, Tween 60, and ergosterol molecules were randomly placed in a box area of 4.50 × 4.50 nm while the hydrophilic heads and the hydrophobic tails located inversely towards each other in the box.

**Figure 2:**
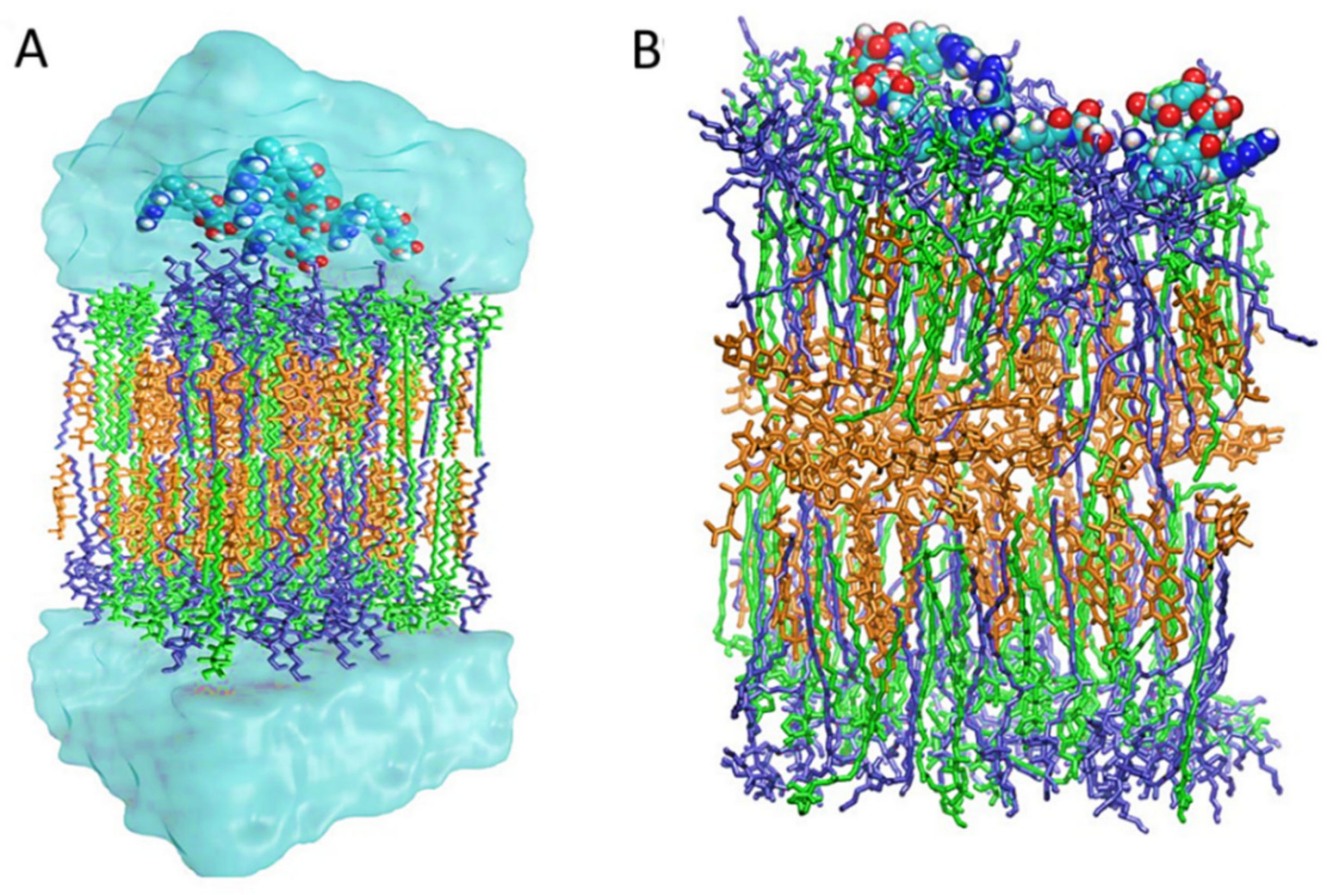
(**A**) The starting structure of niosome bilayer containing MTX at top. Span 60, Tween 60, and ergosterol molecules are colored green, purple, and orange, respectively. The MTX molecules are represented as the space-filling model. (**B**) The final structure of bilayer after 40 ns MD simulation with MTX drugs in contact with bilayer components.

As it is shown in Figure 2, MTX molecules were inserted in the upper leaflet of the bilayer to investigate the interactions of these molecules with the niosome bilayer. The final structure was solvated in an SPC/E water box as this model can reproduce the surface tension of water more accurately [50]. The force field parameters and electrostatic charge distributions of all molecules were obtained from the ATB server [51] based on Gromos87 [52] and Gromos96 54a7 force fields [53]. To avoid the unexpected overlap between atoms in constructed niosome bilayer, the whole system was subjected to energy minimization using the steepest descent algorithm. A short simulation annealing was conducted in 500 K for 100 ps to remove the remaining clashes among atoms in the box. The system was subsequently subjected to NVT simulation run for 500 ps at 298 K to equilibrate the system’s temperature. The choice of this temperature corresponds to optimal conditions for the formation of niosome vesicles that show the highest stability [54]. In this step, Span 60, Tween 60, and ergosterol were coupled together to the v-rescale thermostat with a coupling constant of 0.1 ps, while the water and MTX were coupled separately. The resulted configuration was then subjected to 10 ns of NPT simulation using Berendsenbarostat [55] with semi-isotropic coupling to keep the temperature and pressure of the system constant at 298 and 1 bar, respectively. Periodic boundary conditions were applied in all three directions. Bond constraints were applied using the LINCS algorithm [56], and electrostatic interactions were calculated using the fast particle mesh Ewald (PME) method [57]. The cut-off for coulomb and van der Waal interactions was assigned to 1.5 nm. The systems were simulated for 40 ns production run in Gromacs 2020.1 package.

### 2.4. Morphology of Formulation

The morphology of the prepared pH-responsive niosomal MTX vesicles (before sonication and filtering) was examined using an optical microscope for shape homogeneity and lamellarity (ICC50 W, Wetzlar, Germany).

The particle morphology of the niosomal suspension (after sonication and filtration procedure) was examined using TEM (EM10C, Zeiss, Germany) operating at 120 kV. A proper amount of pH-MTX/Nio vesicles were transferred on a carbon-coated TEM copper grid and left to air dry to form a distributed niosome film. After the grid was inserted in the TEM, multiple images were taken at appropriate magnifications.

### 2.5. Size and Zeta Potential

The DLS technique combined with a diode-pumped solid-state laser was used to characterize the size, PDI, and zeta potential of pH-responsive niosomal MTX. By calibrating the intensity scale with toluene against scattering, DLS was performed at 25 °C and an angle of =90° to the incident ray. Before measuring, the sample solutions were directly filtered into scattering cells using Millipore Millex filters (0.22 μm porosity) and equilibrated for 10 min at the required temperature. The samples’ viscosity was assumed to be that of water [58]. A dielectric constant of 78.5 and a dispersant viscosity of 0.89 cP were used for setup, and all tests were done in triplicate and reported with standard deviation.

### 2.6. Encapsulation Efficiency

The difference between the total amount of MTX introduced in the formulation and that remaining in the aqueous medium after dividing the niosomal suspension by centrifugation at 15,000 rpm for 1 h at 4 °C using a cooling centrifuge was used to calculate the encapsulation efficiency percent (EE%) (5415D, Eppendorf, Germany). MTX concentrations were measured using a UV–visible spectrophotometer at a wavelength of 310 nm (Agilent Technologies, Cary 50, Santa Clara, CA, USA), and EE% was calculated as

(1) $$EE\%=\left[\frac{\left(Total\;amount\;of\;MTX\;added-amount\;of\;free\;MTX\right)}{Total\;amount\;of\;MTX\;added}\right]\times 100$$

### 2.7. In Vitro Release Study

The release behavior of free MTX as control and MTX in pH-responsive niosome in two different PBS mediums (pH 5.4 and 7.4) were performed by dialysis approach. After soaking a dialysis bag with cut-off 12 kDa (Sigma-Aldrich) in pH 7.4 phosphate-buffered saline (PBS), 1 mL of each sample was poured into the dialysis bag. The bags were closed on both ends and positioned in a baker containing 50 mL of PBS with pH 7.4 or 5.4, which were kept at 37 ± 0.1 °C and agitated at 120 rpm. 1 mL of each sample was taken and replaced with fresh release media at the same temperature at specific time intervals. The concentration of each sample was tested using a UV spectrophotometer operating at 310 nm. All of the release studies were carried out in triplicate, with the average values and standard deviation. Different mathematical models were used to examine the MTX release kinetics of loaded formulations (zero order, first order, Higuchi, and Korsmeyer-Peppas) [59].

### 2.8. Cytotoxicity and Morphological Assessment

The monolayer cell culture was trypsinized, and cells (MCF7 and HUVECs) were suspended in DMEM containing 10% FBS. To each well of the 96-well microplate, 200 µL of diluted cell suspension (6 × 10^3^ cell/well) was seeded and allowed to attach overnight. Then, the supernatant was discarded, and 200 µL of escalating concentrations of free MTX, niosomal MTX, and cisplatin (as a reference drug) were added to each well and incubated for 48 h in 5% CO_2_ incubator at 37 °C. At the end of the incubation period, morphological examinations were carried out, and cells were imaged using an inverted microscope with a camera attached (Nikon Diaphot-TMD, Japan). Meanwhile, the cytotoxic activity of free and niosomal MTX was evaluated using MTT colorimetric assay [60]. Free and niosomal-MTX-treated cells were washed with PBS, and 20 µL of MTT reagent (5 mg/mL in PBS) was placed into each well and incubated further for 3 h at 37 °C. In metabolically active cells, MTT is reduced and is converted to insoluble formazan crystals [61]. The precipitated formazan crystals were dissolved in 200 µL of DMSO, and the optical densities (ODs) were read at 570 nm using a SpectraMax microplate reader (Molecular Devices, Sunnyvale, CA, USA). The percentage of viable cells was calculated as ((OD_test_-OD_blank_)/(_ODuntreated cells_-OD_blank_)) × 100. GraphPad Prism software version 6.01 (GraphPad software Inc., San Diego, CA, USA) was utilized to calculate half-maximal inhibitory concentrations (IC50s). This assay was repeated at least three times.

### 2.9. Animal Treatments and Experimental Design

In the current experimental study, 32 white male rats (Wistar breed) were obtained from the laboratory animal breeding colony of the faculty of veterinary medicine, University of Zabol, Zabol, Iran. The mean weight of rats was 221 g. Before the experiments, rats were housed under standard laboratory temperature (25 ± 2 °C), 40% humidity, and 14 h light/10 dark cycles. The rats were allowed to have free access to sterile water and standard rat chow. The experimental procedure and animal handling were conducted following the international guidelines of care and the use of laboratory rodents NIH publication no. 85–23. Before the start of the intraperitoneal injections, the rats were divided into four groups. Rats in the control group received six weeks of intraperitoneal injections of saline. Rats of the second group were received free MTX (2 mg/kg BW). The third and fourth groups received niosomal MTX (2 and 4 mg/kg, respectively). Doses of free MTX and niosomal MTX were selected based on the previous studies and preliminary experiments [62].

Blood samples were obtained by the retro-orbital sinus puncture following light anesthesia 24 h after the last treatment. The collected blood samples were sent to the clinical pathology laboratory of the Faculty of Veterinary Medicine, University of Zabol, and Zabol, Iran. Blood samples were then centrifuged at 5000× *g* for five min to obtain serum samples. Serum samples were kept at −80 °C until the time of experiments. Animal handling and experimental procedure were conducted according to the guidelines of the animal research committee of the University of Zabol, Zabol, Iran (IR. UOZ. REC.1400.0016).

#### 2.9.1. Serum Biochemical Parameters

The serum levels of BUN (blood urea nitrogen) and creatinine were determined calorimetrically according to the Pars Azmoon company protocols (Pars Azmoon. Co., Tehran, Iran). Determination of serum BUN and serum creatinine was performed using the Selectra Pro M autoanalyzer, (Vital Scientific, Amsterdam, The Netherlands). As a measure of lipid peroxidation in rats, serum and tissue malondialdehyde levels were assessed by the Okhawa method with minor changes.

#### 2.9.2. Histopathological Examination

At the end of the study, animals were sacrificed by light anesthesia (1.5% pentobarbital sodium) and the cervical dislocation method. Testis samples were sliced open longitudinally and were preserved in 10% neutral buffered formalin (NBF) for two days. After tissue fixation, the histopathological sections were stained with the hematoxylin-eosin method, periodic acid Schiff method (PAS), and Alcian blue staining method. Finally, tissue micrographs were examined under a light microscope (Tokyo, Olympus, Japan). Other tissue samples were stained by the Alcian blue staining method and were investigated by a light microscope.

### 2.10. Statistical Analysis

The obtained biochemical data were expressed as mean ± SD. The statistically significant difference between experimental groups was determined by using the one-way ANOVA/Tukey post hoc test. All statistical analyses were performed by SPSS software (version 16).

## 3. Results

### 3.1. Physicochemical Properties of Niosomal MTX

**Figure 3:**
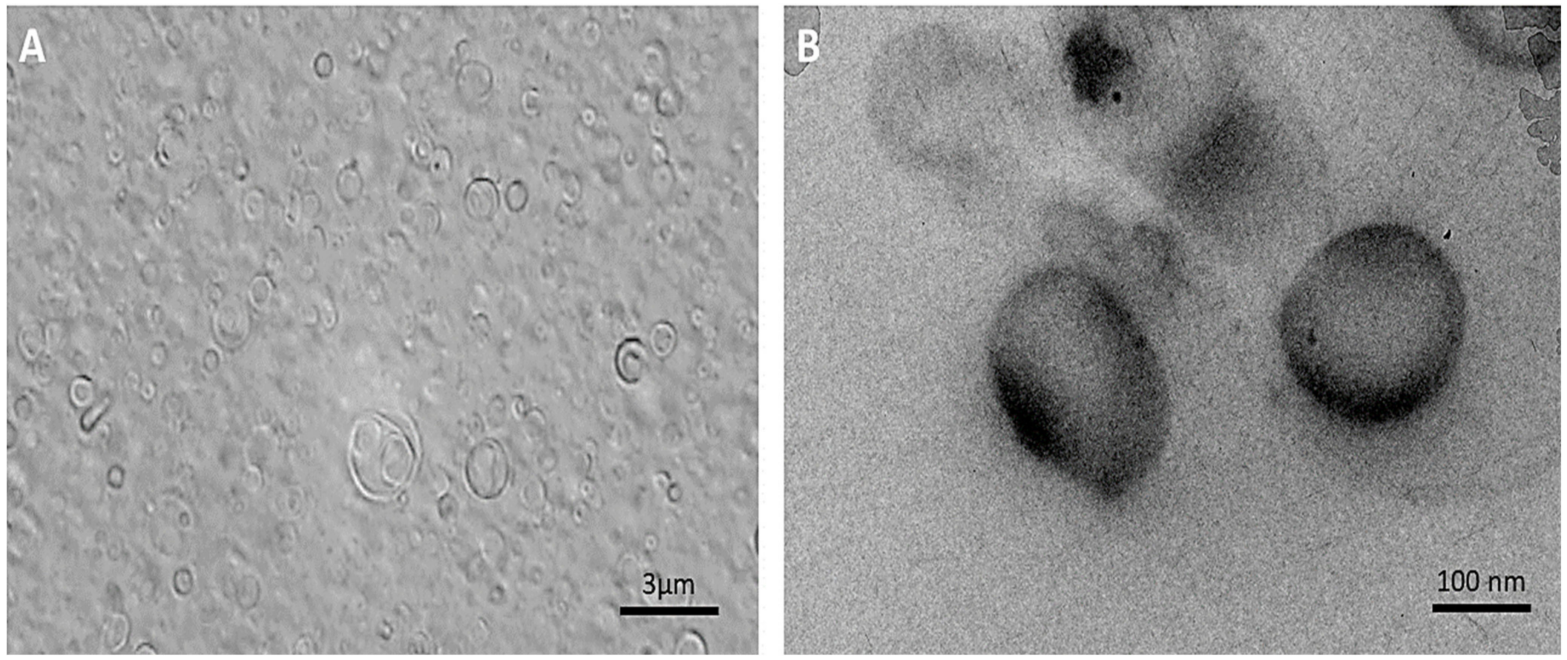
(**A**) Optical microscopy (before ultrasonic and filtration procedure) and (**B**) TEM image (after ultrasonic and filtration procedure) of pH-responsive niosomal MTX.

The morphological appearances of niosomes before sonication and filtration were evaluated by optical microscopy. Based on Figure 3A, pH-responsive niosomal MTX have a spherical shape and multi-layered architecture. Furthermore, transmission electron microscopy (TEM) was used to identify the size and architecture of pH-responsive niosomal MTX (after sonication and filtration). The TEM analysis allowed for a single high-resolution picture to be recorded. Figure 3B shows that pH-responsive niosomal MTX has a size of about 150 nm and a spherical structure.

**Figure 4:**
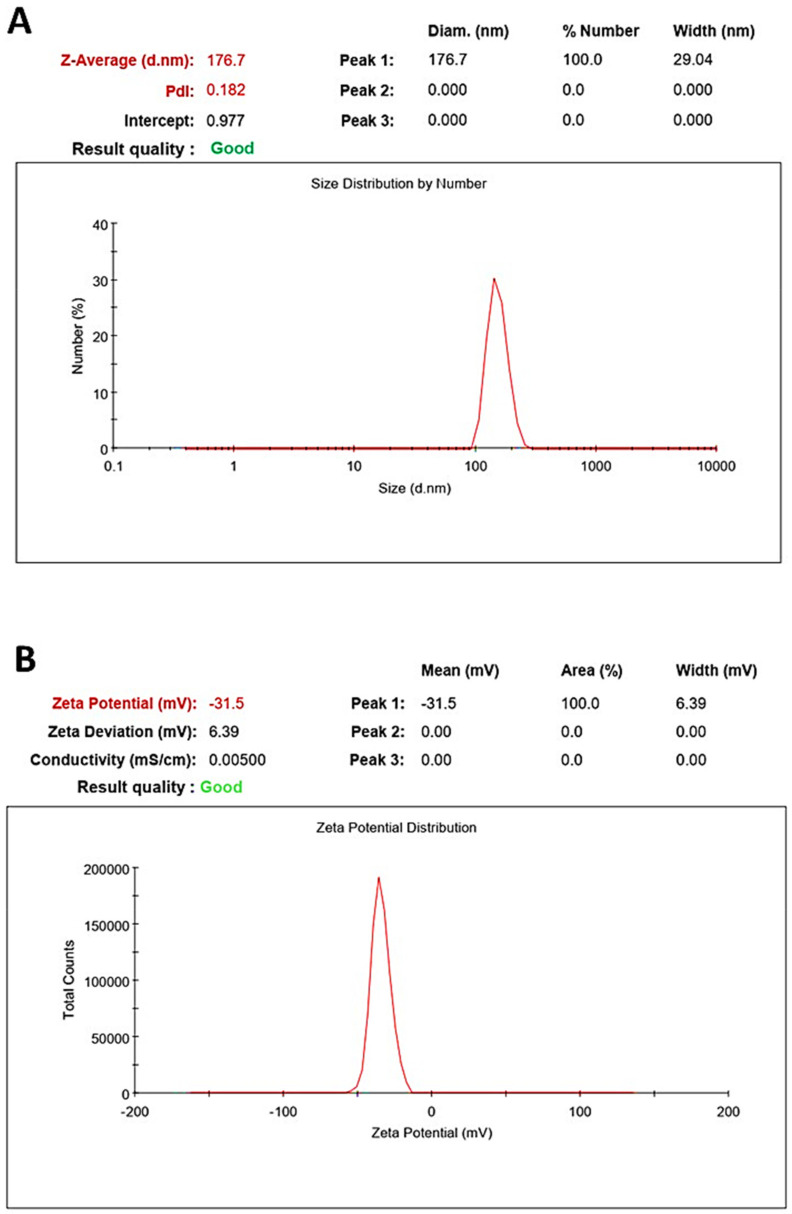
Size distribution, PDI (**A**), and zeta potential (**B**) of pH-responsive MTX-niosome measured by DLS at 25 °C.

The size of vesicles affects their cell penetration and, as a result, their absorption into tissues. It is well documented that particles with a diameter of 100–200 nm have a four-fold higher up-take capacity in tumors than those with diameters of <50 and >300 nm [63,64]. Figure 4A shows the particle size and PDI of the pH-MTX/Nio vesicles. The average size of the formulation was about 176.7 ± 3.4 nm. The polydispersity index, or PDI, is a critical metric for understanding particle behavior and physical stability. The pH-responsive niosomal MTX vesicles were successfully prepared on a monodisperse phase with a PDI of 0.182, resulting in a stable and uniform formulation. The average size of niosomes obtained by DLS was in good agreement with the findings of TEM.

The stabilization quality of vesicle formulations is a goal that can be predicted using zeta potential measurements. According to popular belief, dispersions with zeta potential values greater than 30 mV (negative or positive) are regarded as highly stable. The zeta potential of the synthesized niosomes was about −31.5 ± 2.6 mV (Figure 4B), which proves their stability.

### 3.2. Simulation Results

#### 3.2.1. Bilayer Structure

One of the main properties related to membrane bilayer is area per lipid (APL), by which one can evaluate the phase transition of the lipid bilayer and validate the force field. However, we calculated the APL using the XY surface area of the simulation box divided by the number of Span 60, Tween 60, and ergosterol in one leaflet. Table 1 compares the structural properties, APL, and bilayer thickness at the beginning and the end of simulation time. The average APL for Span 60 is 24.69 ± 0.1 Å2 at the beginning of MD simulation, while this value diminished to 22.26 ± 0.1 Å2 at the end, which has excellent consistency with the experimental value (22 Å2) [65]. Inserting Tween 60 and ergosterol to the Span 60 bilayer closely packed with high order orientation does not expand the bilayer. Tween 60 and ergosterol possessed an average APL of 22.82 ± 0.1 and 26.08 ± 0.1 Å2 after 40 ns MD simulation.

The bilayer thickness is another structural property of the system, which is defined as the mass center of every bilayer component headgroup—i.e., Span 60, Tween 60, and ergosterol. The calculated thicknesses at the beginning and the end of the simulation for each component are represented in Table 1. It is seen that the bilayer thickness for Span 60, Tween 60, and ergosterol were 5.92 ± 0.001, 6.95 ± 0.001, and 2.85 ± 0.001 at the end of the simulation. The GridMAT-MD script [66] was utilized to calculate the APL and thickness of each lipid in niosome bilayer.

#### 3.2.2. Hydrogen Bonding between Membrane and MTX

**Figure 5:**
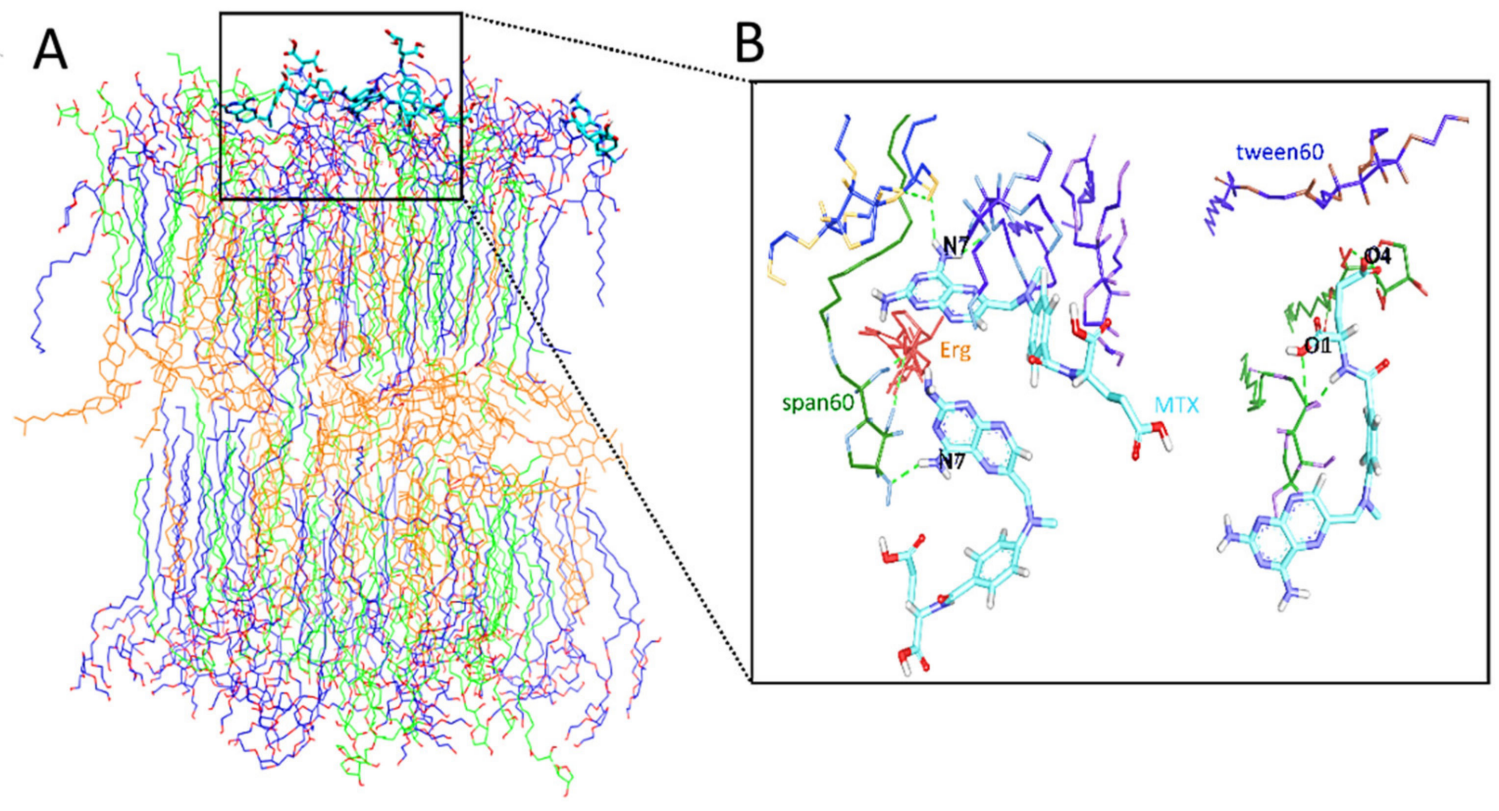
(**A**) Molecular representative structure of MTX at the edge of lipid bilayer as facial representation and (**B**) the close-up representation of most probable hydrogen bonds between MTX and bilayer components. Span 60, Tween 60, ergosterol, and MTX are colored green, purple, red, and cyan, respectively.

In order to determine the stability of hydrogen bonds between three components of niosome bilayer—Span 60, Tween 60, and ergosterol—with MTX as a loading drug, MD analyses of drug-bilayer complex stability were performed during 40 ns of trajectory period. Hydrogen bond profiles between MTX and lipids were calculated using the H-bond utility of Gromacs 2020.1. The threshold for H-bond forming was 3.5 Å with an angle of 30°. Figure 5A illustrates the average position of MTX on one side of the bilayer during 40 ns MD simulation. It is evident from Figure 5A that, in most periods of simulation time, the drugs are close to the edge of the bilayer, where they participated in strong interactions with lipids. Figure 5B represents the site view of MTX contacts with Span 60, Tween 60, and ergosterol. The main interactions observed in this representative structure are N2, N7, and O4 in MTX, making hydrogen bonds with headgroups of Span 60 and Tween 60. More details have been shown in Table 2.

It is apparent from Table 2 that the interaction of MTX with ergosterol (average hydrogen bond of ~0.0002) is much lower than the other components. Due to the further distance between these two species, ergosterol intends to create a hydrogen bond through its head-group with Span 60 and Tween 60 [67]. Table 2 also reveals that the most interaction of MTX with bilayer is through making strong hydrogen bonds with Span 60 headgroup. The occupancy numbers related to the hydrogen bond lifetime between O4, O28, O29, and O30 atoms of Span 60 with N2 and O4 atoms of MTX are evidence for this assertion (Figure 1). Likewise, although hydrogen bonds are less strong than Span 60, noticeable ones still took place between MTX and Tween 60 headgroup. The most important atoms in Tween 60 are O7 and O8, which made strong H-bond with N2, O3, and O1 atoms of MTX (Figure 1).

#### 3.2.3. Radial Distribution Function Analysis

**Figure 6:**
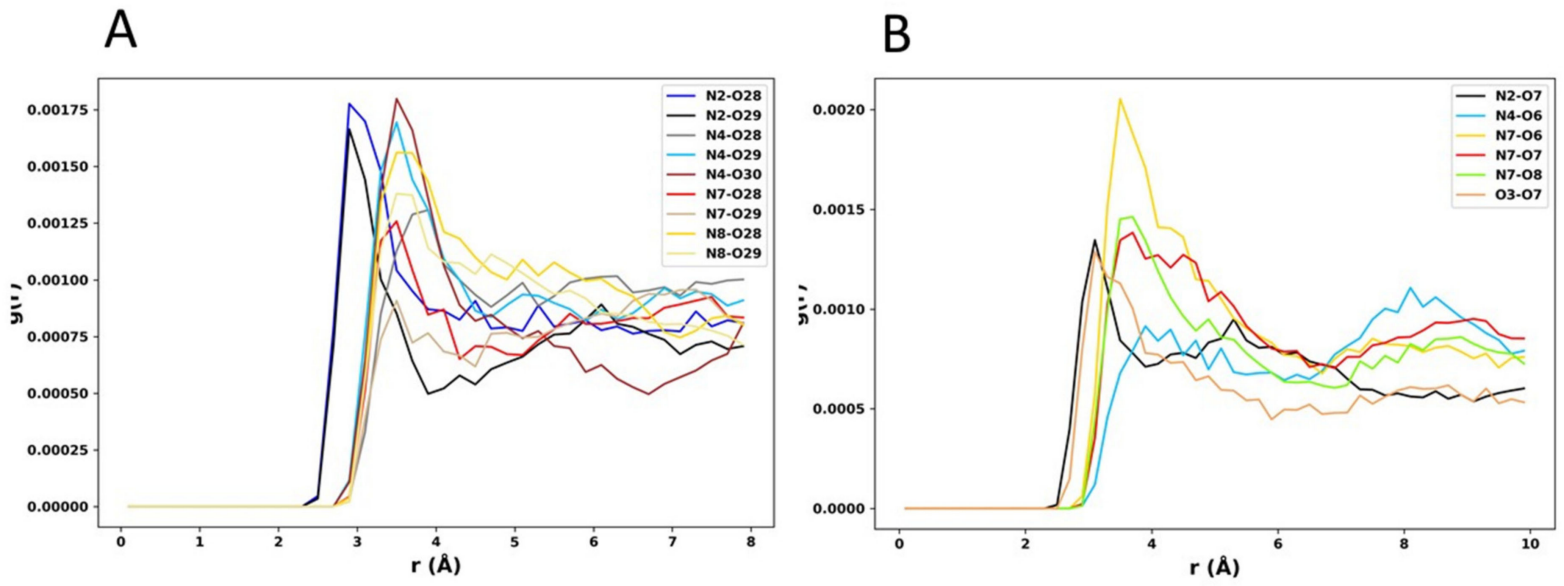
Atom–atom RDFs of (**A**) Span 60 and (**B**) Tween 60 headgroup atoms with MTX atoms (atom numbering corresponds to Figure 1).

Examining the forces that arrange the MTX molecules at the surface of the bi-layer is intriguing. Average radial distribution functions (RDF) plots of MTX, Span 60, and Tween 60 can provide additional structural insight into the interactions involved in creating the MTX-niosome system. Figure 6 presents RDF charts for the system.

The structurally accurate assessment of MTX binding to the niosome bilayer components requires finding the prior arrangement of the molecule towards the bilayer during MD simulation. MTX is composed of a pteridine ring, *p*-aminobenzoic acid, plus glutamic acid. We performed RDF calculations for N2 in glutamic acid and N4, N7, and N8 in pteridine ring towards head group atoms of Span 60 (O28, O29, and O30) to evaluate each part’s arrangement. Based on hydrogen bonding criteria for Tween 60, we also took into account the O3 atom in the glutamic acid moiety of the MTX in RDF calculation. As demonstrated in Figure 6A, the most intense RDF peaks, including N2 interaction in the glutamic acid moiety of MTX with O28, O29, and N4 in pteridine ring moiety of MTX with O29 and O30, are located in 2.8 and 3.8 Å, respectively. This is in line with hydrogen bonding analysis of MTX with Span 60 headgroup, where we observed N2 atom in MTX interacts with O28 and O29 of Span 60 headgroup. According to Figure 6B, the most probable interaction occurs between the N7 atom in the pteridine ring of MTX and the O6 atom of hydroxyl group in one of oxyethylene moieties connected to the five-members ring Tween 60. This peak is located at 3.9 Å, which is beyond the distance we defined for hydrogen bonding. Referring to Figure 4B, there are two packs regarding N2 and O3 in the glutamic acid moiety of MTX interacting with O7 atom of Tween 60 headgroup positioned in 3.0 Å. These interactions are in the hydrogen bond formation range, and hence we can observe them in hydrogen bonding reported in Table 2.

### 3.3. Entrapment Efficiency

The EE% of pH-PTX/Nio formulation was found to be around 76.9% ± 2.5%. With such a high EE%, Span 60 could be an appropriate nonionic surfactant for MTX encapsulation, which is a water-insoluble drug due to its hydrophobic amphiphile composition. On the other hand, Tween 60 is a hydrophilic amphiphile with an HLB value of 14.9. One possible explanation for this EE% is the good interaction of the drug and these surfactants.

### 3.4. In Vitro Release Experiment

**Figure 7:**
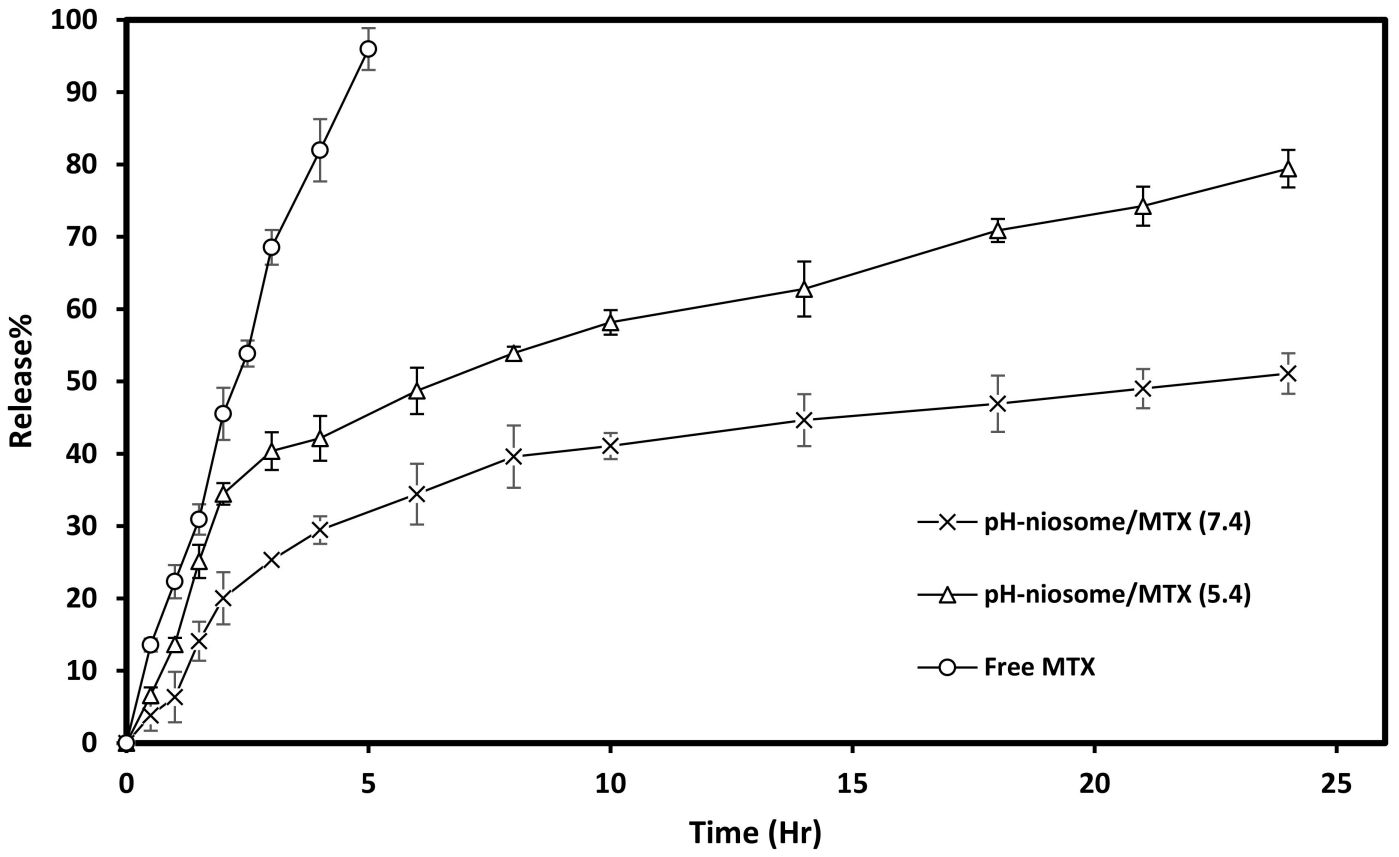
MTX release profiles from free MTX and pH-responsive niosomes in pH 5.4 and 7.4 PBS buffer mediums at 37 °C.

The release studies of MTX from the pH-responsive niosomal formulation in two different PBS solutions (pH 5.4 and 7.4) were performed, and the release profiles are given in Figure 7. It was demonstrated that drug release was completed by 5 h from free MTX at pH 7.4. On the other hand, the pH-responsive niosomal MTX at pH 5.4 seemed to display an initial burst release (42.1 ± 2.8% in 4 h) and a slower release phase up to 24 h (79.4 ± 3.1%). The prepared formulation at pH 7.4 showed a plateau release after 10 h and a sustained release reached 51.2 ± 2.8% after 24 h. The data were statistically analyzed by multiple *t*-tests for each time point. The differences between free MTX and niosome in two pHs (5.4 and 7.4) were considered significantly different (*p* < 0.05).

Table 3 summarizes the results of fitting the in vitro release data to various kinetic models. For both pH values, release data revealed an excellent correlation with Higuchi kinetics. The release exponent (*n*) was found to be in the range of 0.4–0.8, indicating that the anomalous mode of drug transport predominated. The combination of the diffusion and erosion mechanisms characterizes the release mechanism in this case.

### 3.5. Cell-Killing Activity of Niosomal MTX

**Figure 8:**
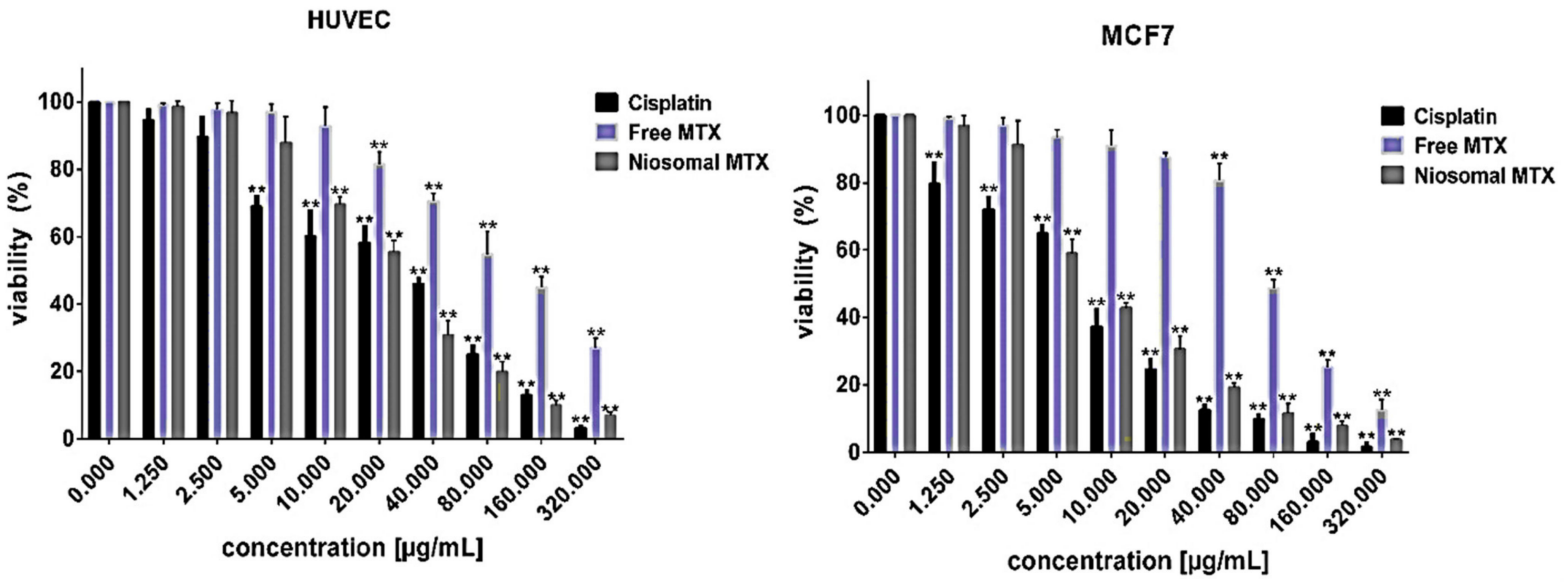
Cytotoxic activity of free- and niosomal MTX on cancerous (MCF7) and normal (HUVEC) cells using MTT colorimetric assay. Cisplatin was used as a reference drug. Results were expressed as the average of three independent (mean ± SD) (** *p* < 0.05 compared with untreated cells).

Cells were exposed to increasing concentrations of the three agents. We observed that exposure to free and encapsulated MTX induces a concentration-dependent reduction in the viability of cancerous and non-cancerous cells (Figure 8). The percentage of viable HUVEC cells following treatment with 0, 1.25, 2.5, 5, 10, 20, 40, 80, 160, and 320 µg/mL of niosomal MTX was 98.7, 96.8, 87.9, 69.6, 55.6, 30.9, 20.1, 10.0, and 7.1%; while these percentages were 96.9, 91.4, 59.0, 42.9, 30.6, 19.3, 11.6, 7.9 and 3.8% for MCF7 cells, respectively. The calculated IC50 values for treating HUVEC cells with free MTX, encapsulated MTX, and free cisplatin were 108.7, 23.14, and 22.67 μg/mL after 48, respectively. However, these values were 84.03, 9.964, and 6.784 μg/mL when MCF7 cells were treated with free MTX, encapsulated MTX, and free cisplatin in the given period.

### 3.6. Morphological Alterations

**Figure 9:**
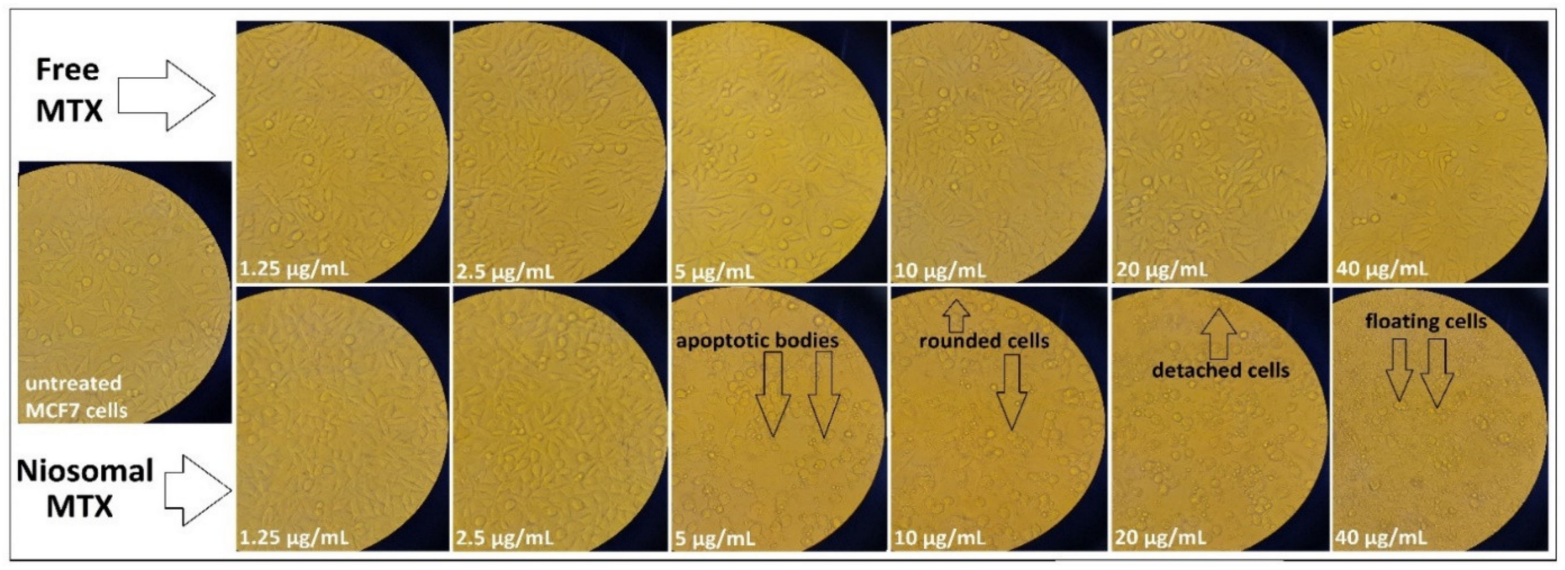
Morphological assessment of MCF7 breast cancer cells treated with 1.25–40 μg/mL of free- and niosomal MTX for 48 h.

We then treated MCF7 cells with increasing concentrations (0, 1.25, 2.5, 5, 10, 20, 40 µg/mL) of free and encapsulated MTX for 48 h. As shown in Figure 9, untreated cells had standard morphology and intact membranes and served as controls. Exposure of MCF7 cells to 1.25–40 µg/mL of free MTX did not induce any morphological alteration in cells, except for a mild decrease in the number of viable cells. In contrast, encapsulated MTX with concentrations higher than 5 µg/mL induced noticeable morphological changes in MCF7 cells, such as cell shrinkage, cell roundness, and formation of apoptotic bodies. When MCF7 cells were treated with high concentrations of niosomal MTX (≥20 µg/mL), cells were detached from culture plates and floated in culture media.

### 3.7. In Vivo Experiments

In the current experimental toxicity study, six weeks of treatment with niosomal MTX and free MTX caused significant increases in serum biochemical parameters and tissue malondialdehyde contents. As shown in Table 4, serum creatinine levels were significantly higher in rats treated with free MTX compared to the control rats (*p* < 0.05). Intraperitoneal treatment with niosomal MTX at 2 mg/kg and 4 mg/kg doses caused significant increases in serum creatinine levels (*p* < 0.01 and *p* < 0.001, respectively). The statistical analysis revealed a significant increase in serum BUN levels of rats treated with free MTX and niosomal MTX compared to control rats (*p* < 0.001). On the other hand, serum urea levels were significantly higher in rats treated with free MTX and niosomal MTX compared to the control rats (*p* < 0.001). Serum MDA levels showed significant increases in rats receiving the 2 mg/kg dose of free MTX compared to the control group (*p* < 0.05). Treatment with niosomal MTX at 2 and 4 mg/kg doses also increased serum MDA levels (*p* < 0.01) for both. Renal MDA levels showed significant increases in kidney lipid peroxidation of rats receiving the 2 mg/kg and 4 mg/kg of niosomal MTX (*p* < 0.5 and *p* < 0.01, respectively).

### 3.8. Histopathological Investigation

**Figure 10:**
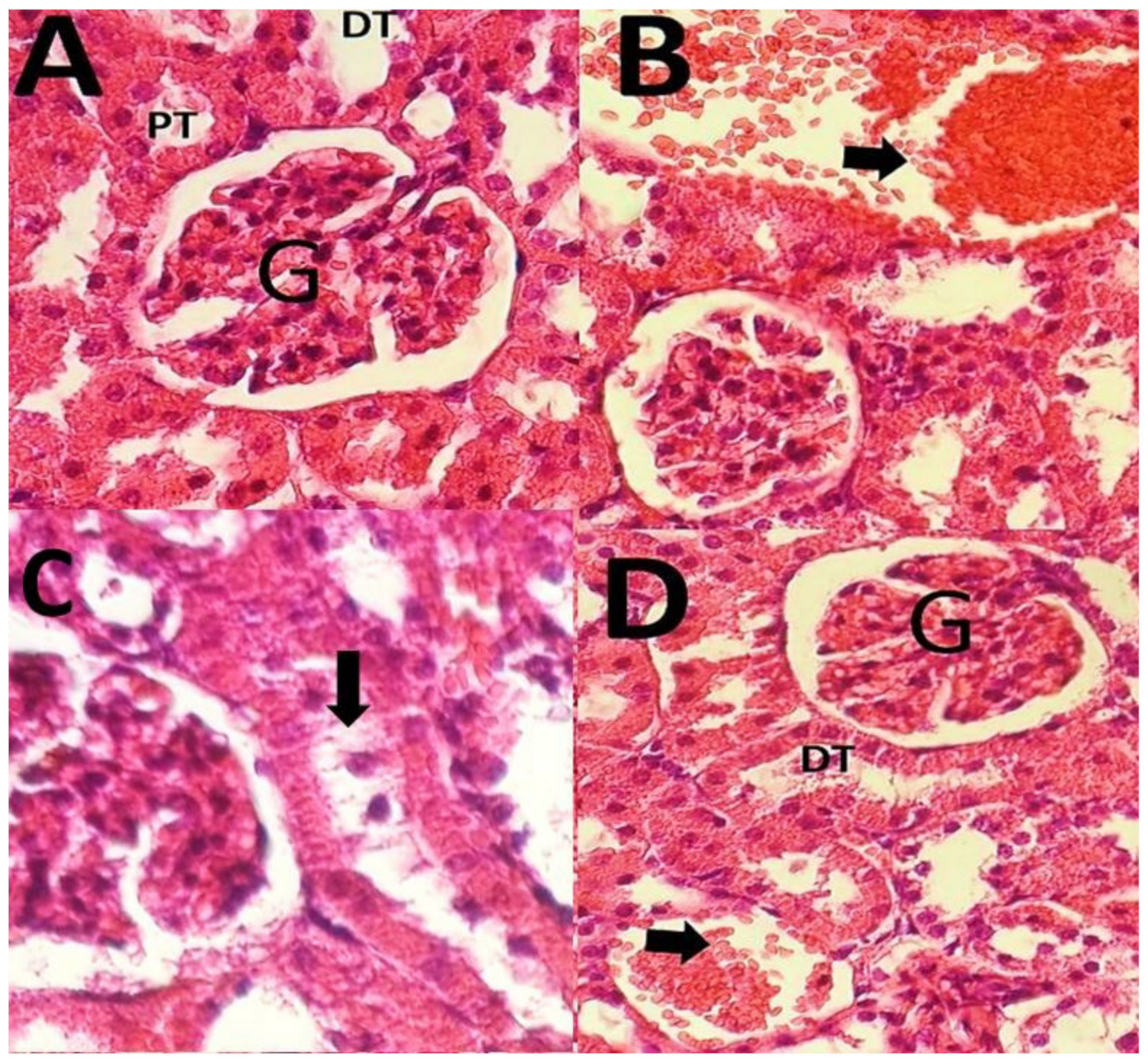
Studying the effects of free MTX and niosomal MTX at a histological level. Hematoxylin-eosin (H&E) staining was done to study hemorrhage (arrows) and cytoplasmic vacuolation (arrow points) in kidney sections. (**A**) Kidney sections of a control rat showing normal glomeruli (G), distal tubule (DT), and normal proximal tubules (PT). (**B**) Kidney sections of a rat that received the 2 mg/kg dose of free MTX revealed severe hemorrhage (arrow) (× 40). (**C**) Kidney section of a rat received the 2 mg/kg dose of niosomal MTX, which shows cytoplasmic vacuolation. (**D**) Kidney section of a rat treated with niosomal MTX reveals hemorrhage (arrow). H&E staining (photomicrographs at × 40).

The histopathological investigations of different experimental groups showed that the rats in the control group had normal kidney structure with normal glomeruli and normal tubules, and normal macula densa apparatus (Figure 10A). The histopathological investigation of the kidney of the group treated with free MTX revealed severe hemorrhage and necrosis (Figure 10B). Histopathological analysis investigation of the group treated with 2 mg/kg dose of niosomal-MTX revealed cytoplasmic vacuolation of proximal and distal tubules (Figure 10C). The microscopic investigation of kidney sections of rats treated with 4 mg/kg dose of niosomal MTX showed necrosis and blood congestion (Figure 10D).

**Figure 11:**
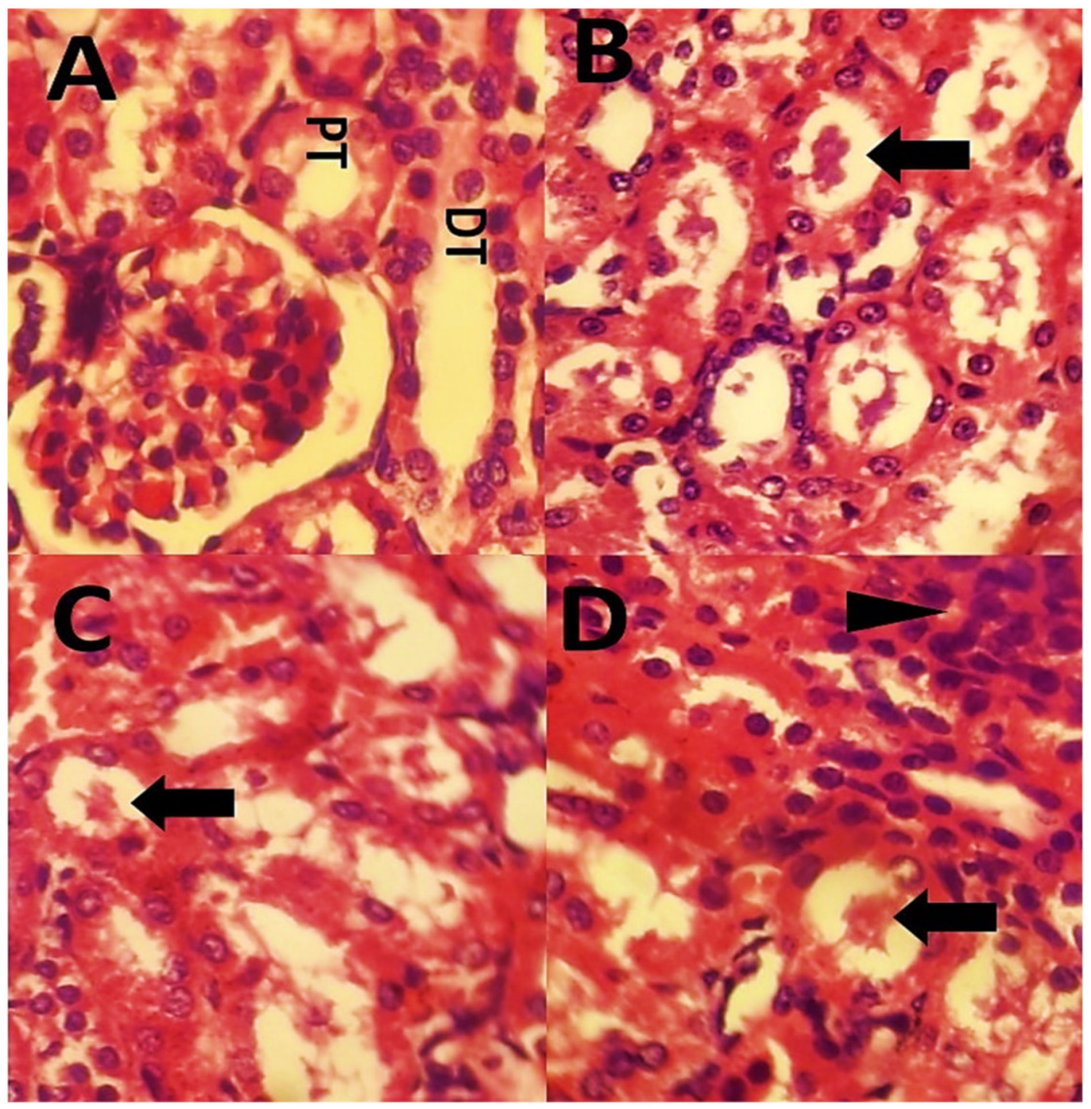
(**A**) Kidney sections of a control rat showing (G) normal glomeruli, normal distal tubules, and normal proximal tubules. (**B**) Kidney sections of a rat that received the 2 mg/kg dose of free MTX reveals whispered hyaline casts in proximal and distal tubules (arrow). (**C**) Kidney micrographs of rats treated 2 mg/kg of niosomal MTX revealed hyaline casts. (**D**) Kidney section of a rat treated with 4 mg/kg dose of MTX, thickening of the wall of kidney tubules (arrow point), and hyaline casts (arrow). Photomicrographs (**A**–**D**) at × 40.

The micrographs of control rats were normal (Figure 11A). Rats treated with free MTX showed severe hyaline casts in proximal and distal tubules (Figure 11B). Kidney micrographs of rats treated 2 mg/kg dose of niosomal MTX revealed hyaline casts (Figure 11C), and the kidney section of a rat treated with 4 mg/kg dose of MTX showed thickening of the wall of kidney tubules (Figure 11D).

**Figure 12:**
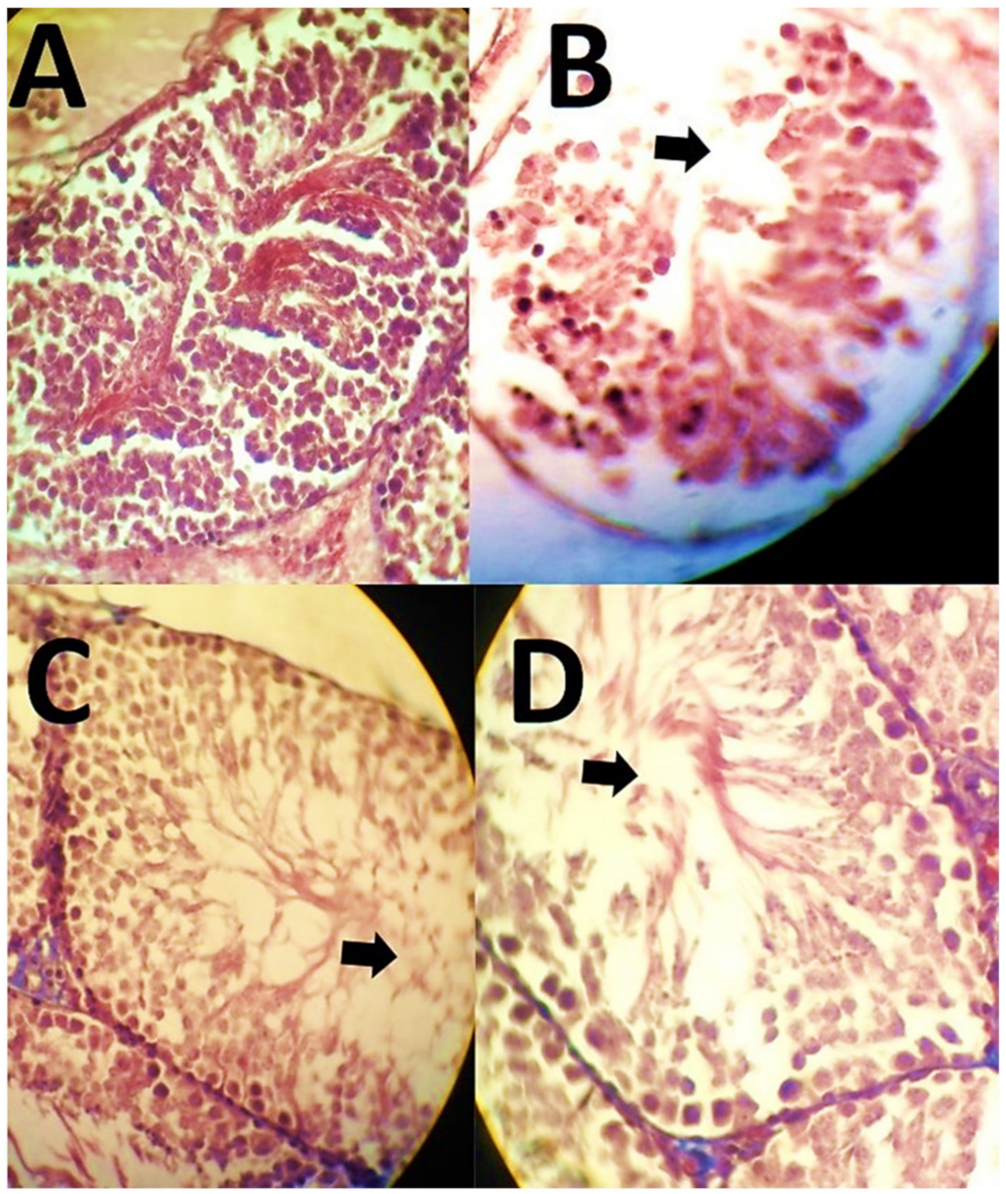
(**A**) Periodic acid-Schiff-stained section of a control rat with normal histopathology. (**B**) Testis sections of a rat treated with free MTX (2 mg/kg). (**C**) Testis micrograph of a rat treated with niosomal MTX (2 mg/kg) reveals degeneration of seminiferous tubules (arrow). (**D**) Testis micrograph of a rat treated with niosomal MTX (4 mg/kg) revealed severe spermatogonia cells reduction. PAS staining, all micrographs at × 40.

Periodic acid-Schiff-stained section of a control rat with normal histopathology (Figure 12A). Testis sections of a rat treated with free MTX (2 mg/kg) showed degeneration of spermatogonium cells (Figure 12B). Testis micrograph of a rat treated with niosomal MTX (2 mg/kg) reveals degeneration of seminiferous tubules (Figure 12C). Testis micrograph of a rat treated with niosomal MTX (4 mg/kg) exhibited a marked reduction in spermatogonia cells (Figure 12D).

## 4. Discussion

In this study, pH-responsive niosomal MTX was prepared by the thin-film hydration method and characterized in terms of physicochemical properties, Computrol simulation, cytotoxicity, and in vivo aspects. Categories of surfactants, surfactant–ergosterol ratio, the concentration of surfactants, hydration solution, molecular weight of active agent, drug concentration, and other factors can influence particle sizes [68]. The appropriate distribution or PDI has values less than 0.4, and a lower PDI value indicates a smaller size distribution, whereas particles in a wide distribution tend to agglomerate [22,69]. Because niosome formation is difficult for chemicals with high hydrophilic−lipophilic balance or high-water solubility in other terms, such as Tween 60 or Span 60, ergosterol is a crucial addition that causes the surfactants to form into niosomes. In recent work, we found that using ergosterol rather than cholesterol resulted in better niosomes [33]. The synthesized niosomes had a zeta potential of −31.5 ± 2.6 mV. Zeta potential is a colloidal stability marker, although it may not reflect the complete picture [70]. Because MTX is negatively charged at physiological pH, it can operate as a charge-inducing agent on its own. It might be attributed to the adsorption of counterions or the preferential adsorption of hydroxyl ions at the vesicle surface. Span 60 and Tween 60 have some functional groups (OH, -ROH, ROR) that have a negative surface charge in water solution. Similar results were reported by Junyaprasert et al. [71], Silva et al. [72], Najlah et al. [73], and many others. The EE% of drug delivery systems is a crucial metric for their effectiveness [22]. In this study, the dialysis procedure was used to determine the EE% of the pH-MTX/Nio formulation [22]. Several studies have found that the results of this approach is similar to that of ultrafiltration, with a few exceptions [74]. For pH-responsive niosomes, a high EE% of about 76.9% was obtained. Because MTX is insoluble in water, it is potentially positioned in hydrophobic tail groups. As a result, MTX could be found in both the hydrophobic tail groups and the aqueous interior of niosomes. This microenvironment could help MTX find a better location in niosomes. To put all of these data into context, the EE% in niosomal vesicles is influenced by lipid concentration, type of surfactant, and surfactant concentrations.

Furthermore, numerous researchers have observed that using mixed niosomes with two different surfactants can help MTX dissolve better in a drug carrier system. For example, Demirbolat et al. examined single surfactant niosomes and mixed niosomes with the same surfactant: cholesterol concentrations and same aqueous solvent and observed that the mixed niosomes had higher EE% [30]. Release data also showed an excellent response to the different pH. The pH sensitivity of the formulation came from CHEM in the bilayer. Since CHEMS has a cholesterol-like structure, it can stabilize the phospholipid bilayer under neutral conditions, and because it has a special steroidal rigid structure, it exhibits better pH response characteristics under acidic conditions [75].

Also, the APL and thickness of the niosome bilayer were evaluated by MD simulation. Based on results, the APL of Span 60 reached 22.26 ± 0.1 Å2 at the end of simulation which has an excellent consistency with the experimental value. Inserting Tween 60 and ergosterol to the bilayer model with aver-age APL of 22.82 ± 0.1 and 26.08 ± 0.1 Å2, respectively, does not affect the bilayer’s expansion. In addition, the main interactions of MTX with the lipid bilayer components were investigated. Results indicated that the permanent interaction of MTX with the bilayer is through making strong hydrogen bonds with the Span 60 headgroup. MTX intended to make a strong hydrogen bond with Span 60 compartments involving N2 and O4 atoms in glutamic acid and N7 atoms in pteridine ring moieties, respectively. Based on results from MD simulation, the Tween 60 head group made the weaker interaction with MTX through N2, O3, and O1 atoms in the glutamic acid moiety. RDF analysis was conducted, which can give us additional structural insight into the interactions involved in the formations of MTX-niosome system. According to this investigation, the atomic RDF curves regarding N2 interaction in the glutamic acid moiety of MTX with headgroup atoms in Span 60 is in line with hydrogen bonding analysis. The RFD carves in the case of Tween 60 suggested that the most probable interaction occurs between N7 atom in the pteridine ring of MTX and O6 atom of hydroxyl group connected to a five-member ring of Tween 60.

The cytotoxic activities of free and encapsulated MTX in malignant and non-malignant cells were compared by different tests. Results showed that niosomal MTX exhibited a safer profile in normal human cells than free cisplatin (higher IC50s), whereas in malignant cells, free cisplatin-induced better cell-killing effects than free and niosomal MTX (lower IC50s). As for niosomal MTX, higher cytotoxic effects were observed in cancer cells, while normal cells were more resistant to this formulation (IC50 = 9.464 and 23.14 µg/mL in MCF7 and HUVEC cells, respectively). This is important since novel drug-developing strategies are aimed to minimize the adverse drug side effects on healthy cells/organs [76]. Morphological examinations showed that compared with free MTX, encapsulated MTX exerted a distinct set of morphological changes mainly observed during apoptotic cell death. Still, more investigations are needed to elucidate the primary cell death mechanism activated by our formulation. It was previously shown that vesicle formation reduces surfactant toxicity. Hence, many niosomal formulations were introduced as drug delivery systems (DDS) for cancer therapy [77]. Using topical MTX-loaded niosomes with safe profiles was previously suggested to improve the management of psoriasis, an inflammatory skin disorder of unknown origin [78]. In cancer therapy, efforts were put to improve the efficacy of existing anti-neoplastic agents by encapsulating them in niosomes [79,80]. Most studies on MTX encapsulation have focused on entrapping this drug in nanomicelles [81] and nanoliposomes [82]. There are few reports on the efficacy of niosomal MTX against cancer cells.

In this respect, Sheena encapsulated the hydroxypropyl-β-cylodextrin complex in niosomes using Span 60, cholesterol, and dicetyl phosphate. This resulted in enhanced MTX solubility, and the prepared nanoniosomes exhibited better stability and improved anticancer activity in tumor-bearing BALB/c mice [83]. Two years later, Oommen et al. developed a DDS by successful entrapment of cyclodextrin-MTX complex in niosomes. Their findings indicated that the entrapped complex exhibited an improved anticancer activity in C57BL/6J mice bearing melanoma B16F1 tumor [84]. Most recently, Demirbolat and colleagues used sodium hydroxide as a hydrating solution for synthesizing small-size MTX-loaded niosomes. Their observation revealed that the solubility of the formulation was enhanced. Simultaneously, the number of dead cells, mainly due to activation of apoptotic cell death, was markedly increased in treated cells, and MTX-loaded niosomes were found to be safe [30]. In agreement with the findings of these reports, our in vitro results suggest that newly prepared nano-niosomes exerted desirable anticancer effects and could be considered a novel DDS in cancer therapy.

Finally, both niosomal formulations and the free form of MTX showed toxic effects on the kidney and testis of rats. Previous studies have demonstrated that free-form MTX can easily cross the blood testis barrier and induce severe histological changes in the testis [85]. In previous studies, chronic or sub-chronic administration of MTX resulted in a decrease in spermatogonium cells, a decrease in the diameter of seminiferous tubules, and significantly increased interstitial spaces [86]. Our results showed that prominent histological alterations were observed in the testis that indicates reproductive toxicity of niosomal MTX. In the current work, histopathological changes such as necrosis were also observed in the seminiferous tubules of rats treated with niosomal MTX, suggestively due to an effect of the niosome components on the permeability of the blood-brain barrier. These histopathological changes were similar in groups treated with 2 mg/kg and 4 mg/kg doses of MTX. It could be concluded that the reproductive toxicity of niosomal MTX is not dose-dependent. Some studies have shown that niosomal MTX seems to reduce the rapid formation of 7-hydroxy methotrexate compared to the free form of MTX [87], while some other studies suggested the increased toxicity of niosomal MTX [88]. Our results showed that niosomal MTX could have some toxic effects at some doses.

Our study has some limitations. First, we did not assess cell death by performing fluorescent and nonfluorescent cytosolic and nuclear staining techniques or flowcytometric determination. Second, we did not evaluate gene expression changes in cell death pathways. Furthermore, cytotoxicity and undesirable side effects of the prepared formulation were only tested on two cell lines and needed further examination. For in vivo study, determination of lipid peroxidation and antioxidant enzyme activity were the limitations of the current study.

## 5. Conclusions

The prepared pH-responsive niosomal MTX had a nanometric range with high stability. EE% of 76.9% and responsive pH behavior proved the potential ability of nanocarrier in medical application. Based on simulation results, the MTX molecule interacts mainly with head groups of lipid bilayer compartments due to its glutamic acid moiety, where it forms strong hydrogen bonds with Span 60 head-group. Our formulation had desirable cytotoxic effects against cancer cells. Niosomal MTX and free MTX showed toxic effects on the kidney and testis of rats. Still, the safety profile of such a formulation in healthy cells/tissues should be further investigated

## Figures and Tables

**Table 1:** Comparison of the structural properties of Span 60, Tween 60, and ergosterol bilayers in 35:35:30% mol at the beginning and the end of MD simulation.

Lipid	Span 60		Tween 60		Ergosterol	
Time	0 ns	40 ns	0 ns	40 ns	0 ns	40 ns
Area per lipid (Å^2^)	24.69 ± 0.1	22.26 ± 0.1	25.31± 0.1	22.82 ± 0.1	28.92 ± 0.1	26.08 ± 0.1
Thickness (nm)	5.78 ± 0.001	5.92 ± 0.001	6.03 ± 0.001	6.95 ± 0.001	2.75 ± 0.001	2.85 ± 0.001

**Table 2:** Average and detailed hydrogen bond analysis of MTX with Span 60, Tween 60, and ergosterol in this study. SPN: Span 60; TWN: Tween 60; ERG: ergosterol; MTX: methotrexate.

			**Hydrogen Bonds**			
	**MTX- Span 60**		**MTX- Tween 60**		**MTX-Ergosterol**	
**Average hydrogen number**	0.56		0.31		0.0002	
**Detailed hydrogen bonds**	**Donor-** **Acceptor**	**Occupancy**	**Donor-** **Acceptor**	**Occupancy**	**Donor- Acceptor**	**Occupancy**
	MTX@N2- SPA@O28	10.92	MTX@N2- TWN@O7	3.22	ERG@O1-MTX@N6	0.02
	MTX @N2- MTX@O29	8.82	TWN@O7- MTX@O3	2.47		
	SPN@O28- MTX@O4	3.72	TWN@O7- MTX@O1	2.05		
	MTX@N2- SPN@O30	3.27	MTX@N2- TWN@O8	1.80		

SPN: Span 60; TWN: Tween 60; ERG: ergosterol; MTX: methotrexate.

**Table 3:** Kinetic models of MTX released from pH-responsive niosomes in pH 7.4 and 5.4.

Kinetic Model	pH	Equation	R^2^
Zero order	7.4	y = 0/0295x + 15/827	0/7866
	5.4	y = 0/043x + 24/525	0/8413
First order	7.4	y = 0/0005x + 1/1188	0/5358
	5.4	y = 0/0005x + 1/3419	0/5599
Higuchi	7.4	y = 1/4093x + 2/69	0/9163
	5.4	y = 2/0138x + 6/1851	0/9427
Korsmeyer and Peppas	7.4	y = 0/6226x − 0/1416	0/8873
	5.4	y = 0/5503x + 0/2331	0/8884

**Table 4:** Effects of free MTX and niosomal MTX on biochemical parameters and kidney MDA content of different experimental groups.

Parameter	Treatment *			
	Control	Free MTX 2 mg/kg	Niosomal MTX 2 mg/kg	Niosomal MTX 4 mg/kg
Creatinine (mg/dL)	0.79 ± 0.16	1.1 * ± 0.10	1.2 ** ± 0.25	1.32 *** ± 0.4
BUN (mg/dL)	11.1± 3.3	26.6 *** ± 3.6	23.6 *** ± 7.0	24.1 *** ± 6.1
Urea (mg/dl)	5.1 ± 1.4	19.2 *** ± 3.2	25.0 *** ± 7.8	24.3 *** ± 3.6
Serum MDA (nmol/ml)	141.6 ± 34.0	189.1 * ± 33.6	200.1 ** ± 37.3	206.3 ** ± 21.1
Kidney MDA(nmol/mg)	212.6 ± 22.9	298.8 * ± 82.5	269.6 * ± 49.7	318 ** ± 63.4

MTX: Methotrexate; BUN: blood urea nitrogen. * significant for the control group (*p* < 0.05). ** significant for control group (*p* < 0.01). *** significant for control group (*p* < 0.001).

## Data Availability

Data are included within this article.
